# The role of Herceptin in early breast cancer

**DOI:** 10.1186/1477-7800-5-9

**Published:** 2008-04-28

**Authors:** Ashok Subramanian, Kefah Mokbel

**Affiliations:** 1 St Georges Hospital, Blackshaw Road, Tooting, SW17 0QT, UK; 2 Brunel Institute of Cancer Genetics, Uxbridge, Middlesex, UK

## Abstract

Herceptin is widely regarded as the most important development in the treatment of breast cancer since Tamoxifen and the development of the multidisciplinary team (MDT). It is particularly exciting from an oncological polint of view as it represents success in the emerging field of specific targeted therapies to specific molecular abnormalities in tumour cells. This review will focus on the nature of the Her2 overexpression and the role of herceptin in the treatment of early breast cancer.

## Introduction

### The structure of HER-2

**Figure 1:**
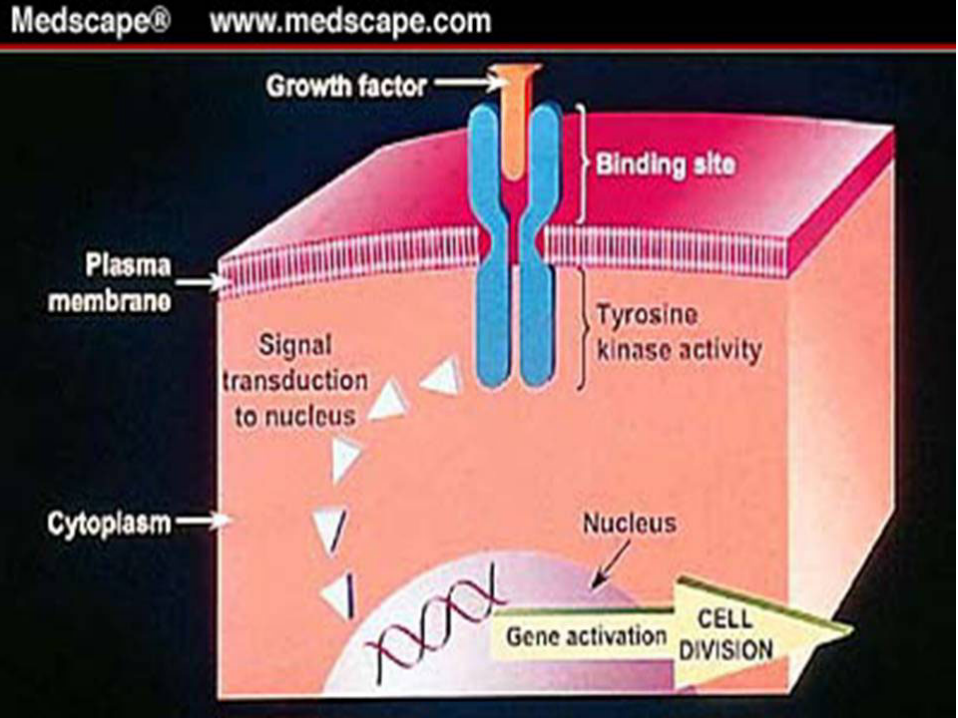
The HER2 receptor dimer transmembrane signal transduction pathway (with kind permission of Roche).

The HER-2 (neu/cerB2) proto-oncogene is located on chromosome 17 and encodes a 185 kDa transmembrane tyrosine kinase receptor which exhibits extensive homology to the epidermal growth factor receptor [1-5]. It consists of an extracellular domain, a transmembrane domain and a cytoplasmic tyrosine kinase domain via which it exerts it's intracellular action (figure 1).

It appears that the receptor is constitutionally active but on interaction with it's ligand [6,7], heterodimerisation occurs activating the receptor further [8-11].

### The role of Her-2 in health and disease

In normal cells, HER-2 plays a key role in cellular growth factor signal transduction and is also involved in the regulation of cell growth, survival and differentiation [12]. The Her-2 oncogene can be activated by point mutations, gene amplification or over-expression and it is now well accepted that this predicts for a poor outcome in mammalian breast cancer [13,14] and is associated with ER negativity and nodal/brain metastasis. Her-2 overexpression occurs in approximately 20–30% of breast cancers [15] and is also overexpressed in lung, ovarian and gastric adenocarcinomas [16].

HER2 over expression has also been found to be an independent prognostic predictor of overall survival and time to relapse [15,17,18]. Several authors have reported a higher frequency of HER2 over expression in ductal carcinoma *in situ *(DCIS) compared with invasive cancer [19,20] Similarly, Liu *et al *[20] have observed HER2 overexpression as determined by gene amplification using polymerase-chain reaction (PCR)-based techniques in 48% of *in situ *carcinomas compared with 21% of stage II invasive breast tumours. These results were also confirmed using immunohistochemical techniques (IHC). Allred *et al *[19] reported HER2 over expression as measured by IHC in 56% of cases of pure DCIS (77% in comedo lesions), in 22% of infiltrating ductal carcinomas (IDC) associated with DCIS and 15% of IDC not associated with DCIS. None of the hyperplastic/dysplastic breast lesions overexpressed HER2. These observations suggest that HER2 plays a significant role in the genetic initiation of mammary carcinogenesis rather than in disease progression [21]. HER2 expression also appears to show good concordance between the primary tumour and both synchronous and subsequent metastasis in both the intensity and pattern of IHC staining [22,23] suggesting that the oncogenic overexpression remains stable.

## Clinical Her-2 Testing

Accurate measurement of HER2 amplification and/or overexpression is vital due to the prognostic and potential therapeutic implications of being HER-2 positive.

Two main methods exist for the determination of HER2 gene amplification and protein expression in breast cancer specimens, fluorescence in situ hybridisation or FISH (direct or indirect) and IHC. The former measures gene amplification in breast cancer specimens whilst the latter measures protein expression.

In the FISH technique, fluorescence labelled cDNA probes for HER2 (chromosome 17 q11.2–q12.0) and chromosome 17 centromeres [(chromosome enumeration probe 17 (CEP17)] are used. The HER2 gene appears as a red/orange signal and the CEP17 appears as a green. A ratio of HER2:CEP17 copy number > 2 denotes amplification when taken over an average of at least 60 invasive cancer cells.

In the IHC technique, membrane staining of malignant cells is assessed using the appropriate antibody in fixed tumour blocks. It is a semi-quantitative technique with the intensity of staining reflecting the amount of protein present. From the therapeutic standpoint, the recommended scoring system to evaluate IHC staining is shown in Table 1 below.

**Table 1:** The IHC scoring system of HER2

**Staining pattern**	**Staining score**
Membrane staining is observed in < 10% of the tumour cells	0
A partial and faint membrane staining is detected in > 10% of the tumour cells	1+
A weak to moderate complete membrane staining is detected in > 10% of the tumour cells	2+
A moderate to strong complete membrane staining is detected in > 10% of the tumour cells	3+

Those tumours which score 0 or 1+ are regarded as negative, those scoring 2+ are borderline and 3+ are positive.

This system has been used in pivotal trials [24] evaluating the efficacy of a humanised anti-HER2 monoclonal antibody therapy in woman with advanced breast cancer and has been approved by the appropriate authorities in the USA and the EU.

**Figure 2:**
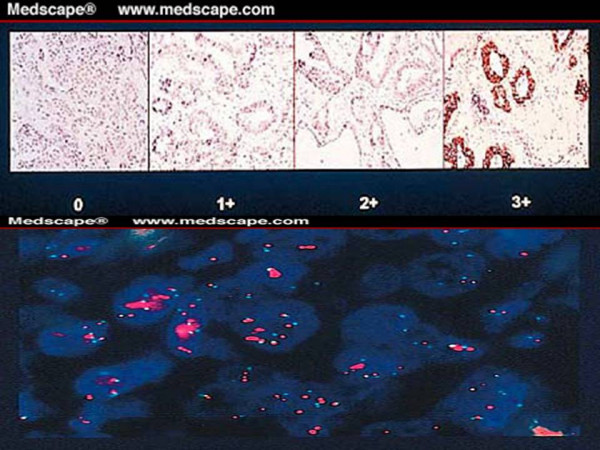
HER2-positive testing (a) by IHC (courtesy of Dc M. J. Kornstein, Medical College of Virginia) and (b) by FISH.

Ridolfi *et al *(23) tested 750 consecutive invasive carcinomas for HER2 overexpression using both IHC and FISH techniques. He found that whereas the concordance rate between FISH and IHC (positive = 3+, negative = 1+, 0) was 98.7%. FISH was positive in 36% of specimens scored 2+ by IHC (Figure 2) which would have been scored negative by this technique. A pictorial comparison between the two testing methods is shown in figure 2[25].

Hoang *et al *[26] also reported a high concordance rate for HER2 positivity between IHC score 3+ and FISH (89%). However, there was a low interobserver reproducibility in separating 2+ from 3+cases. This data suggests that IHC is a useful initial test and cases scoring 2+ should be considered for FISH testing.

Mass from Genetech [27] recently presented the concordance rates between FISH and the clinical trial assay (CTA), a immunohistochemical technique in 623 samples randomly selected from the two pivotal Herceptin trials. FISH positivity was observed in 4.2%, 6.7%, 23.9% and 89.3% of CTA 0, 1+, 2+ and 3+, respectively.

Conflicting results regarding the best antibody to use in determining the IHC status of HER2 were presented by Falo *et al *[28]and Bartlett *et al *[29], respectively. The former found that monoclonal antibody CB11 was more reliable than the Dako polyclonal antibody compared against FISH, whereas Bartlett reported a higher accuracy for the polyclonal antibody (87.4%) compared with the monoclonal (83.8%). Gancberg *et al *[30] studied the sensitivity of three frequently used antibodies and found that the monoclonal antibody TAB250 had the lowest misclassification rate against FISH in 160 breast cancer specimens.

In view of such conflicting results and the significant inter-observer variation IHC should be performed in standardised reference laboratories with a large caseload. In the United Kingdom, all laboratories performing IHC as a predictive test must participate in an external quality assurance scheme and guidelines recommend that they should be performing at least 250 assays per year (100 for Her2 FISH testing. Alternatively, FISH testing could be performed as the gold standard. The latter option may need to be introduced gradually until the technique and required expertise are established in breast cancer centres.

FISH has been described as not only being both more accurate in determining HER2 status but also is a better predictor of prognosis and response to Herceptin. In addition, DNA is more stable than protein, interpretation is easier and there is less inter-operator error. FISH however is both more labour intensive and expensive.

For these reasons in the United Kingdom, a two-phase testing regimen exists for assessing HER2 status. Initial IHC testing of 0 or 1+ is reported as negative with 3+ being reported as positive. A score of 2+ is reported as intermediate with these cases being referred for FISH to establish a definitive diagnosis.

## The clinical applications of Her2 testing

### Does HER2 Overexpression Predict Adjuvant Tamoxifen Failure in Patients With Breast Cancer?

Tamoxifen is a selective oestrogen receptor (ER) modulator and has been proven to reduce relapse, death rates and risk of contralateral breast cancer and as such is probably the first truly targeted therapy for treating breast cancer [31]. Tamoxifen failure therefore is an area that generates intense research.

Both experimental and clinical evidence have indicated that the HER2 pathway interacts with the ER pathway with retrospective clinical studies suggesting an inverse relationship between ER and HER-2 expression. The proportion of patients with ER/HER-2 +ve breast tumours is approximately 9%.

### Preclinical Evidence

Experimental evidence has shown that oestrogen-dependent MCF7 cells that over express HER2 are rendered tamoxifen resistant and have reduced numbers of ER [32,33]. The promoter of the HER-2 gene also contains an oestrogen response element which suppresses HER-2 expression on response to oestrogen and is overexpressed with tamoxifen [34,35]. Transfection of MCF-7 cells with the HER-2 coding region has also been shown to render them tamoxifen resistant [36]. For these reasons, the HER2 pathway has been investigated as a potential contributor towards Tamoxifen resistance and HER2 has been proposed as a potential marker of Tamoxifen sensitivity.

It appears that the mechanism for this resistance may rest in feedback through joint downstream signalling mechanisms. It has already been described that HER-2 transfected MCF-7 cells are tamoxifen resistant but HER-2 overexpression/stimulation leads to ER downregulation, increased phosphorylation and also increased transcriptional activation [37-39]. In addition, HER-2 downregulation appears to shut down HER-2 initiated MAP kinase pathways and makes other ER-linked apoptotic pathways dominant [40]. Supporting this, blocking MAP kinase in these HER-2 +, ER + cells restores their sensitivity to tamoxifen. Another feature of interest is that MEKK1, a downstream Her-2 signalling mediator activates ER and potentiates the agonist effect of tamoxifen [41]. In this way, HER-2 positivity may act to convert tamoxifen from anantagonist to agonist in breast cancer cells.

### Clinical Evidence

Many clinical studies have found an association between HER2 overexpression and Tamoxifen failure in metastatic breast cancer [42-45] and also a reduced response duration and survival duration in those treataed with adjuvant hormonal therapies. The GUN trial [46], revealed that HER-2 expression not only predicted tamoxifen resistance but also showed a worse outcome on tamoxifen compared to those who were untreated (possibly because of the heightened agonist action). Recently a meta-analysis of seven studies[47] concluded that metastatic breast cancer overexpressing HER2 was very likely to be resistant to Tamoxifen (odds ratio of disease progression, 2.46). Recent work presented at the San Antonio breast cancer conference however did not find such an association. In a large cohort of stage 2 breast cancer patients randomised to recieve either 2 years of tamoxifen or nothing, no difference in tamoxifen efficacy was seen in Her2 -ve patients when compared to those who were Her 2 +ve.

It still remains controversial however as to whether adjuvant Tamoxifen has beneficial or detrimental effects in early breast cancer over expressing HER2 [48,48-51]. Five of six studies so far have shown that treating patients who are HER2 positive with adjuvant Tamoxifen does not have a beneficial effect, and Bianco *et al *[52] have even reported a detrimental effect.

Among patients receiving adjuvant Tamoxifen, a combined analysis [53] was undertaken of studies with analysable data [48,51,52,54] using the number of patients and the length of median follow up as a weighting factor for the relative risk of relapse or death per study. This revealed that HER2 over expression was associated with an increased the risk of relapse/death by 75% (RR = 1.75). Such results strongly suggest that over expression of HER2 predicts reduced response to adjuvant tamoxifen in patients with EBC, but does not exclude benefit in patients with HER2 and ER/PgR-positive tumours.

Available clinical evidence, although limited, suggests that the response to aromatase inhibitors in HER-2 positive breast cancers is superior to tamoxifen. In randomised studies of ER +ve HER-2 +ve patients treated with either Tamoxifen or Letrazole, there was a good response in the letrazole arm compared to a negligible response with Tamoxifen [55,56]. It is postulated that this may be due to the fact that aromatase inhibitors effectively reduce the amount of active oestrogen rendering the ER monomeric and inactive, whilst the agonist activity of Tamoxifen can still be activated by MEKK1.

### The role of Herceptin in early breast cancer

Herceptin [31] is a chimeric IgG monoclonal antibody (95% human, 5% murine) developed from the murine 4D5 antibody. It targets the external moiety of the Her2 receptor and prevents activation of the protein (possibly by preventing dimerisation) thereby preventing proliferation of breast cancer cells overexpressing Her2.

### Pre-clinical Trials

In pre-clinical studies, anti HER2 MABs have been shown to inhibit the growth of HER2 over expressing tumour cells.

Harwerth *et al*, [57] looked at the effects of HER2-specific MAB administration on the tumorigenic growth of human HER2 transformed NIH3T3 cells implanted into athymic nude mice. Two antibodies (FWP51 and FSP77) inhibited the onset of tumour growth, and led to retardation of growth of established tumours. They were also effective in the treatment of transformed tumours established from SKOV3 cells [58]. Furthermore, the authors observed that the combination of the two antibodies, which react with two distinct regions of the HER2 receptor, was more effective than treatment with either MAB alone.

Hudziak *et al*, [59] showed that a MAB directed against the extracellular domain of p185 HER2 specifically inhibited the growth of breast tumour derived cells over expressing the HER2 gene product. They also showed that resistance to the cytotoxic effect of tumour necrosis factor alpha (a consequence of HER2 overexpression) was significantly reduced in the presence of the antibody.

### Clinical trials

The initial trials regarding the use of Herceptin were concentrated on it's role in the treatment of metastatic breast cancer with anthracyclines, paclitaxel and doxetaxel. We will not be elaborating on this further but instead will concentrate on the evidence for it's use in EBC.

In the neoadjuvant setting, a preliminary small randomised trial showed an excellent pathological CR rate (67%) when Herceptin was used with a number of chemotherapeutic agents (particularly paclitaxel) followed by combination anthracycline treatment when compared with standard chemotherapy alone (25%) [60]. Following these remarkable results, 4 major trials were started recruiting 13,000 patients to assess the role of Herceptin further. Each trial used Herceptin for 1 year either in combination with or following chemotherapy and each has shown a recurrence risk reduction of approximately 50%. The two USA based trials (NSABBP B-31 and NCCTG N9831) combined their data showing benefit when Herceptin was used with paclitaxel following 4 courses of anthracycline [61]. The HERA trial showed similar results when Herceptin was used alone following standard chemotherapeutic regimens with a 46% recurrence risk reduction, 51% distant recurrence risk reduction and a 24% reduction in mortality (not significant), which was seen across all subsets [62]. Recent subgroup analysis has shown this relapse reduction to be independent of the nodal status or hormone receptor profile even in those patients with a relatively low risk of relapse. The BCIRG 006 trial showed similar benefit with docetaxel following 4 courses of anthracyclines [63]. This trial also showed benefit with a novel non-anthracycline schedule using upfront herceptin with docetaxel and carboplatin which would avoid anthracycline related cardiotoxicity. This latter schedule however is probably only as effective as anthracyclines in the 65% of patients whose tumours do not overexpress topo-isomerase 2, a key target of anthrocycline chemotherapy.

Maturation of these trials has also shown a significant survival benefit at 2 years followup of 34% (HERA) which is mirrored by the two American trials.

Although these results are undoubtedly very exciting it should be remembered that the followup is still relatively short. The optimal duration of Herceptin treatment is also subject of considerable debate. A 2 year extended treatment arm of the HERA trial is currently underway with results expected whilst a small Finnish trial has suggested a mere 9 week upfront course of Herceptin may have the same benefit as the larger trials [64].

### The role of herceptin with adjuvant hormonal therapy

It has already been established that herceptin is an extremely useful adjuvant therapy in the HER-2 +ve, ER/PR -ve patient and also that it predicts for tamoxifen resistance. It would therefore seem reasonable to expect that administering Herceptin plus Tamoxifen to ER+ve HER-2 positive patients may overcome this Tamoxifen resistance. This has been proved preclinically using the parent antibody of Herceptin mAb 4D5 [36]. Several other cell culture studies have revealed greater growth inhibition using a combination of Herceptin and Tamoxifen on ER+ve/HER-2+ve cell lines when compared with each agent in isolation [36,65] and have also showed that this is only the case with strongly ER+ve cell lines [66]. Several studies are currently underway investigating this phenomenon further.

### The cost of Herceptin

Herceptin is not without complications with mild to moderate adverse effects occuring in approximately 50% of patients. The most common adverse reactions are related to the initial infusion particularly fever, chills, pain, vomiting and headache [24,24,67], and the most serious adverse reaction is class III/IV cardiac dysfunction. The NSABBP B-31 trial showed an increase in cardiotoxicity of 3.3% in the Herceptin vs the control arm, and the HERA trial produced a 1.7% increase in cardiac failure [68].

Cobleigh *et al *[69,70] reported cardiac dysfunction in 10 patients (4.7%) nine of whom had received anthracycline therapy. There was one cardiac-related mortality. Such data suggest that the combination of anthracycline and herceptin should be avoided and the left ventricular ejection fraction should be measured in patients at risk. Herceptin-related cardiac dysfunction varies in severity and should be treated with standard medical therapy (diuretics, glycosides, ACE inhibitors, etc) and discontinuation of herceptin therapy should be considered when the risks outweigh the benefits.

Other adverse reactions include hypersensitivity reactions and anaphylaxis (rare), pulmonary events including dyspnoea, bronchospasm and ARDS (rare), haematological toxicity (leucopenia, thrombocytopenia and anaemia) and hepatic and renal toxicity. Many of these side effects are believed to be related to the 5% murine component located at the FAB end of the antibody. There have been no reports so far of the development of measurable antibodies to Herceptin inpatients who received the recommended dose.

Herceptin should be avoided in pregnancy as the teratogenic risk is as yet unknown and should also be avoided in breastfeeding and for 6 months afterwards.

Financially, treatment with herceptin and a taxane will cost approximately £20,000 per patient assuming that the treatment is stopped ater 18 weeks which does not include costs associated with testing, and investigation and treatment of cardiac morbidity.

## Conclusion

Herceptin has emerged as the single most important treatment for breast cancer in both the metastatic and neoadjuvant settings, since the emergence of Tamoxifen. It is particularly exciting since it represents the first truly effective targeted treatment to molecular abnormalities in tumour cells. Although the trials are of relatively short followup duration, the treatment is associated with rare but significant side effects and the treatment is not without cost, Herceptin has undoubtedly made a real difference for those 25–30% of patients who overexpress the Her2/neu oncogene. Table 2 summarises the current place for Herceptin in early breast cancer.

**Table 2:** The role of herceptin in early breast cancer

**ER**	**PR**	**HER-2**	**Treatment**
+	+/-	-	Tamoxifen/AI
+	+/-	+	AI
-	+	-	Tamoxifen
-	-	+	Herceptin + chemo
-	-	-	Nothing

In the future the role of combined Herceptin and hormonal therapies will hopefully become more evident which may further benefit this group of patients.
